# Predicting the clinical trajectory of feeding and swallowing abilities in CHARGE syndrome

**DOI:** 10.1007/s00431-023-04841-4

**Published:** 2023-02-17

**Authors:** R. Onesimo, E. Sforza, V. Giorgio, D. Rigante, E. Kuczynska, C. Leoni, F. Proli, C. Agazzi, D. Limongelli, A. Cerchiari, M. Tartaglia, G. Zampino

**Affiliations:** 1 Center for Rare Diseases, Department of Woman and Child Health and Public Health, Fondazione Policlinico Universitario A, Gemelli-IRCCS, 00168 Rome, Italy; 2 Università Cattolica del Sacro Cuore, Largo Vito 1, 00168 Rome, Italy; 3 Pediatric Unit, Fondazione Policlinico Universitario Agostino Gemelli-IRCCS, 00168 Rome, Italy; 4 Feeding and Swallowing Services Unit, Dept. Neuroscience and Neurorehabilitation, Bambino Gesù Children’s Hospital-IRCCS, 00168 Rome, Italy; 5 Genetics and Rare Diseases Research Division, Ospedale Pediatrico Bambino Gesù IRCCS, 00168 Rome, Italy

**Keywords:** CHARGE syndrome, CHD7, Deglutition disorders, Nutritional support, Rare diseases, Paediatrics

## Abstract

To date, the feeding and oral-motor abilities of patients with CHARGE syndrome (CS) have not been longitudinally assessed. This study aims to investigate the level of these abilities at different ages and evaluate how they evolve during growth. We retrospectively analysed oral-motor features of 16 patients with molecularly confirmed CS (age range 4–21 years old; mean 11 years; SD 6 years; median 10 years). Nearly 100% of CS new-borns had weak sucking at birth, and half of them demonstrated poor coordination between breathing and swallowing. Over time, the percentages of children with tube feeding dependence (60% at birth) faced a slow but steady decrease (from 33% at 6 months, 25% at 12 months, to 13% at school age) in tandem with the decreasing risk of aspiration. The ability of eating foods requiring chewing was achieved at school age, after the acquisition of an adequate oral sensory processing. A mature chewing pattern with a variety of food textures was not achieved by more than half of patients, including those requiring artificial enteral nutrition. Most patients started prolonged oral-motor treatments with speech language therapists in early childhood.

*Conclusions*: Although feeding and swallowing disorders are constant features in CS patients, a slow and gradual development of feeding abilities occurs in most cases. Rehabilitation plays a key role in overcoming structural and functional difficulties and attaining appropriate eating skills.

**Table:** 

**What is Known:** *• Feeding problems and swallowing dysfunction have been noted in CHARGE syndrome*. *• The involvement of multiple factors, including structural problems in the mouth, throat, or esophagus, and neurological impairment, make feeding a complicated task in CHARGE individuals*.	
**What is New:** *• Dysphagia gradually improves in most CHARGE children over time, though with a wide interindividual variability*. *• The percentages of children with tube feeding dependence decrease over time from 60% at birth to 33% at 6 months and 13% at school age*.

**Supplementary Information:**

The online version contains supplementary material available at 10.1007/s00431-023-04841-4.

## **Introduction**

CHARGE syndrome (CS) (OMIM #214,800) is a rare congenital disorder with an incidence that ranges from 0.1 to 1.2/10,000 live births [1]. The acronym ‘CHARGE’ describes the constellation of cardinal features characterizing the disorder, including coloboma (very frequent), heart defects (frequent), choanal atresia (frequent), retardation of growth (frequent) and/or development (very frequent), genitourinary malformation (very frequent for male, frequent for female) and external ear abnormalities (very frequent) [2–4]. The visual system is invariably affected, with an association between hypomorphic variants and milder ophthalmological features [5].

Minor diagnostic criteria include orofacial cleft (frequent), distinctive facial appearance, tracheoesophageal fistula (occasional), limb abnormalities (occasional) and rarely immune deficiencies [4, 6].

CS was first recognized by Hall and Hittner and, hence, it was initially called Hall-Hittner syndrome. The acronym CHARGE was first suggested by Pagon et al [7]. CS is an autosomal dominant disorder caused by loss-of-function variants in CHD7 or deletions of the gene within chromosome region 8q12 [8–10], and previous studies reported the detection of pathogenic variants in 70–90% of clinically diagnosed CS cases [11].

Feeding problems and swallowing dysfunction have been reported as a common feature in CS, and over 90% individuals need artificial nutrition during their life [12, 13]. According to Stromland et al., children with CS may experience persistent drooling (25%), resulting in a further negative factor on feeding abilities [14, 15]. Prematurity, neurological impairment, long-term tube feeding and limited experiences with oral intake often result in a limited progression of child’s oral motor skills [16, 17]. Specifically, the ability to suck, swallow or chew can be ineffective or absent due to cranial nerve dysfunction [16]. Moreover, surgical procedures to repair structural anomalies, including cleft lip/palate and choanal atresia, can postpone the introduction of oral feeding [12]. In the majority of cases (> 80%), magnetic resonance imaging demonstrated the absence or hypoplasia of olfactory bulbs and sulci with a consequent or completely absent sense of smell [12]. Poor oral intakes may have unfavourable outcome on bone mineralization and growth during childhood [4, 12] and severe gastroesophageal reflux may imply the placement of a gastrostomy tube and the consequent exclusion of mouth feeding [12, 15].

Although feeding problems and swallowing issues have already been investigated in CS [12], there is no clear understanding of the skill progression according to age, and longitudinal data are lacking. Given the clinical relevance of these aspects, here, we retrospectively collected the data on feeding and swallowing abilities that had systematically been recorded in a relatively large, single-centre CS cohort to evaluate the evolution of the feeding and swallowing abilities with age.

## **Methods**

Retrospective analysis of the clinical records of patients under the age of 21 years with molecularly confirmed CS followed at the Rare Disease Unit, Paediatrics Department, Fondazione Policlinico Agostino Gemelli-IRCCS, Rome, between 2011 and 2021 allowed to identify 16 subjects (8 M; age range 4–21 years; mean 11 years; DS 6 years; median 10 years), all reported to have feeding difficulties.

The Local Ethical Committee approved the study as part of a large protocol evaluation on disability and nutritional aspects in rare diseases patients. The 16 eligible patients were enrolled in the study after signed informed consents were secured.

Findings about multidisciplinary assessment were anonymously collected, including data on oral-motor evaluation of feeding and swallowing abilities, performed as a standard of care.

The comprehensive feeding and swallowing assessment was routinely conducted in accordance with the World Health Organization's (WHO) International Classification of Functioning, Disability and Health (ICF) framework [18]. It considered the congenital abnormalities affecting the swallowing function as part of the case history (i.e. review of clinical records, family interviews), the structural and functional observation of oral-facial structures (i.e. strength, coordination of movement) and information on child eating or being fed by a caregiver. Typical developmental skills (i.e. sucking, chewing), behavioural factors, neurologic functioning and airway protection were also included in the evaluation process. Moreover, data on mean duration of mealtime experience reported by parents of orally fed CS children along with the effectiveness of parent/caregiver and infant interactions, appetite and parental concerns about feeding were also regularly collected. Information of food textures (puree, soft solid, hard solid) tolerated by each child was systematically collected. Assessment included the same questions and procedure for each participant.

This resulted in the classification of patients having oral feeding and swallowing issues or with typical development of feeding skills. If available, we collected objective data from instrumental swallowing assessment including the Videofluoroscopic Swallow Study (VFSS) [19] and radionuclide salivagram [20]. We also contacted each caregiver to clarify missing information.

Descriptive statistics were performed on demographic and clinical characteristics in the data set. Results are presented as mean ± standard deviation, range or percentage.

## **Results**

### Genotype

All subjects carried de novo variants in *CDH7*, including nine truncating mutations (five nonsense and four frameshift), two missense and five nucleotide changes affecting splice sites (Tables 1 and 2). The four children with persistent worse outcome (long-term enteral feeding dependent with or without tracheal cannula) had truncating mutations, while a relatively milder phenotype, which improved with age, was observed in the subjects with splice site changes.

**Table 1:** Details of our cohort of patients with CHARGE syndrome

Patients (number) 16	
Demographics at the time of our study	
Age range (years)	4–21
Median age (years), SD age (years)	10, ± 6
Gender (M)	8
Genetics	
*CDH7* mutation	16
Frameshift	4
Nonsense	5
Missense	2
Splicing	5
Major features (%)* n*	
Choanal atresia/stenosis	(19) 3
Cranial nerve dysfunction	(75) 12
Ocular coloboma	(81) 13
Ear abnormalities	(100) 16
Minor features (%)* n*	
Cardiovascular malformations	(56) 9
Retardation of growth	(81) 13
Retardation of development	(100) 16
Cleft palate	(25) 4
Oesophageal Atresia and/or tracheoesophageal fistula	(12) 2
Tracheomalacia	(6) 1
Genitourinary malformations	(37) 6

*SD* standard deviation

**Table 2:** Genetic variants and persistent worse outcome of our cohort of 16 patients with CHARGE syndrome

	**Genetic variants**					**Enteral nutrition (months)**				
**Pt no**	Nucleotide and protein change		Exon	Intron	Variant type	1	2–6	7–24	> 24	Long-term tracheostomy
**1**	CHD7 c.2509_2512delCATT	p.His837ValfsTer5	8		F					
**2**	CHD7 c.5722_5723delAC	(p.Thr1908ProfsTer17)	29		F	+	+	+	+	+
**3**	CHD7 C.2957 + 5G > A			11	S					
**4**	CHD7 c.1774delC	(p.Gln592SerfsTer16)	3		F	+				
**5**	CHD7 c.4795C > T	(p.Gln1599Ter)	21		NS					
**6**	CHD7 c.3004C > T	(p.Gln1001Ter)	12		NS	+	+	+		
**7**	CHD7 c.6936 + 2 T > A			32	S	+				
**8**	c.969-975delAACAA	(p.Val323TyrfsTer11)	2		F	+	+	+	+	+
**9**	CHD7 c.5782C > T	(p.Gln1928Ter)	29		NS	+				
**10**	CHD7 c.1163C > G	(p.Ser230Ter)	2		NS					
**11**	CHD7 c.3156 T > A	(p.Ser1052Arg)	12		MS	+	+			
**12**	CDH7c.6955C > T	p.Arg2319Cys	33		MS	+				
**13**	CHD7 c.7156-4A > G			33	S					
**14**	CDH7c.2442 + 5G > A			6	S					
**15**	CHD7c.7803C > G	p.Tyr2601Ter	35		NS	+	+	+		
**16**	c. 4644 + 1G > A			20	S					

*MS* missense, *NS* non sense, *FS* frameshift, *S* splicing

### Phenotype

Among the features linked to feeding and swallowing dysfunction, choanal atresia/stenosis and cranial nerve dysfunction were found respectively in 3 (20%) and in 12 patients (75%). Development and growth retardation were detected in all cases; orofacial cleft was detected in 4 (27%); oesophageal malformations included atresia, tracheoesophageal fistula (TEF) or both defects in 2 (12%) and tracheomalacia in one (7%) (Table 1). Percentages of features linked to feeding and swallowing difficulties are reported in supplementary Table 1 with previously reported findings.

### *Feeding and swallowing abilities in new-borns (Table **3**; Fig. **1**)*

**Table 3:** Achievement of feeding abilities by age in the cohort of patients with CHARGE syndrome

		*Our cohort (n* = *16)*	
		***Not adequate for age***	***Not achieved with time***
Milestones [21]		% (*n*)	% (*n*)
New-born	*Full oral feeding*	56 (9)	12 (2)
	*Suckling ability*	100 (16)	
	*Coordination between breathing and swallowing*	50 (8)	
6 months	*Weaning*	31 (5)	12 (2)
12 months	*Start eating solid food requiring chewing ability*	100 (16)	19 (3)
	*Adequate taste division during meal*	100 (16)	
24 months	*Mature chewing pattern*	100 (16)	56 (9)
	*Adequate oral-sensory processing*	93 (15)	19 (3)
	*Adequate mealtime duration*	100 (16)	

**Fig. 1:**
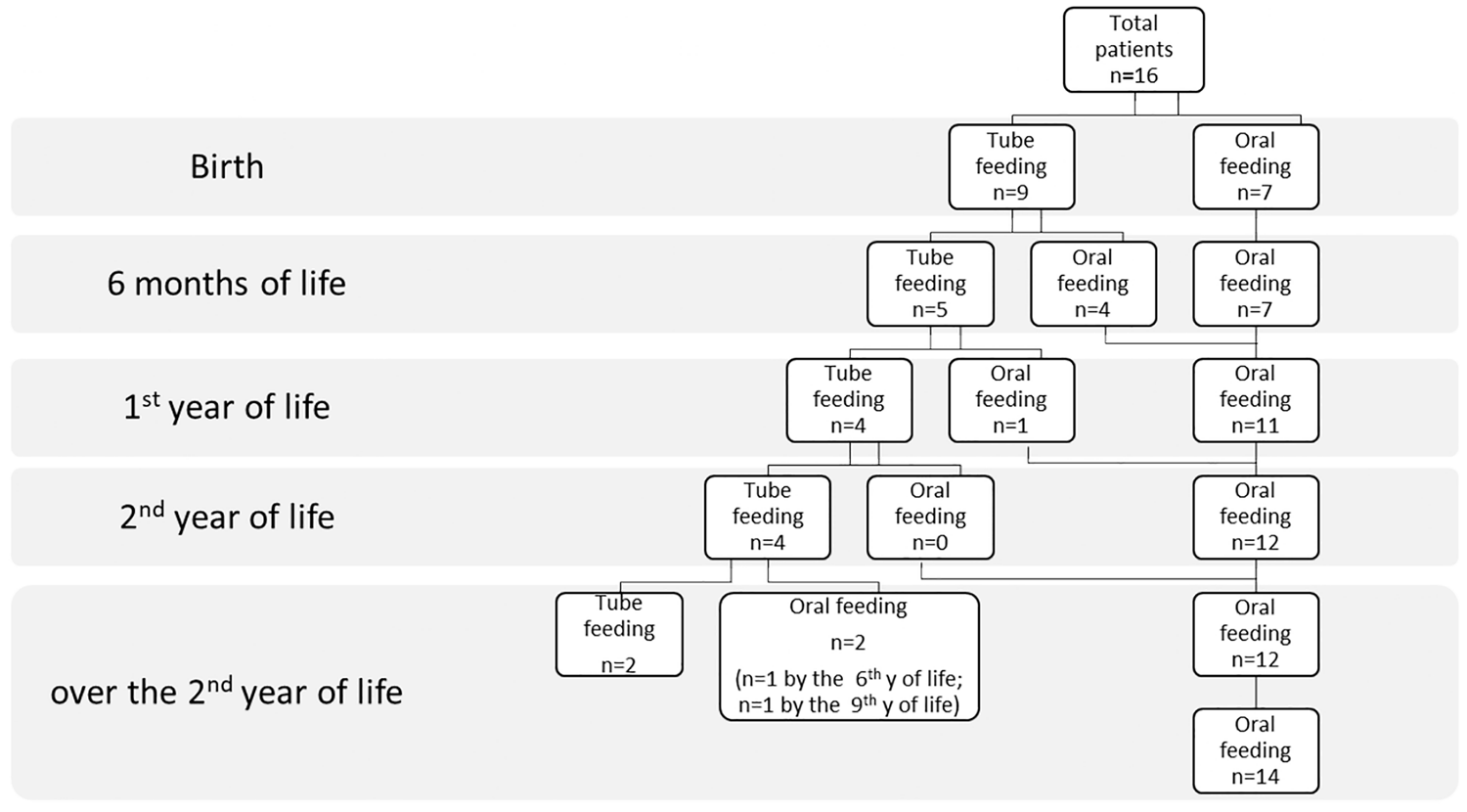
Diagram showing feeding pathway of our cohort of patients with CHARGE syndrome at different ages

Parents reported that 100% of new-borns (*n* = 16) had weak suckling at birth, with half of them demonstrating poor coordination between breathing and swallowing.

Almost 30% of new-borns (*n* = 9) received total enteral nutrition (TEN), specifically through a nasogastric tube (NGT).

### Feeding and swallowing abilities from 1 to 6 months of life

Weaning was completely performed in 11/16 (68%) infants. Moreover, up to 50% of caregivers reported that their children showed food aversion and had extended meal duration.

Within the first 6 months of life, four out of nine infants (44%) were fed by NGT at birth, gradually reaching complete oral feeding.

At this age, 31% of infants (5/16) were still tube feeding-dependent, and about 19% (3/16) had severe aspiration risk, as indicated by salivagram testing.

### Feeding and swallowing abilities from 6 months to the 1st year of life

By the age of 1 year, 25% of children (4/16) still necessitated TEN, and three of them underwent tracheostomy for chronic respiratory insufficiency. The latter presented with airway malformations: one patient presented with cleft palate, the second one presented cleft palate and TEF, and the third presented with tracheomalacia. All three children suffered from severe dysphagia and consequent bolus aspiration due to cranial neurological symptoms and structural defects.

Before 12 months of life, no child started eating soft solid food. At this stage, clinical evaluation displayed multiple factors affecting feeding, including oral-motor and sensory function delay in almost all patients often accompanied by lack of posture control and, more occasionally, by lack of tooth eruption. A constant parental apprehension regarding feeding issues, with concerns on the poor growth status, was noticed.

### Feeding and swallowing abilities from the 1st to the 2nd year of life

By the age of 2 years, a mature chewing pattern was not achieved by anyone. Twenty-five per cent of patients (4/16) still needed TEN via G-tube, as mouth feeding attempts were unsuccessful and accompanied by indirect signs of bolus aspiration.

Food aversion continued to be mentioned by 30% of caregivers, and mealtime duration still remained a major issue at this time.

### Feeding and swallowing abilities over the 2nd year

Thirteen of the sixteen patients (81%) started early prolonged oral-motor treatments with speech language therapists (SLT) in order to improve swallowing function and minimise the risk of malnutrition, dehydration and aspiration pneumonia.

Among the sixteen patents, four still needed TEN via G-tube. Among them, two children gradually became total oral feeders, by the age of 6 and 9 respectively, when VFSS was performed, and safety of swallowing was confirmed. In both cases, it took almost 2 years to reach a complete weaning with constant SLT rehabilitation.

By the age of 7 years, 19% of patients (3/16) still needed TEN via G-tube and still experienced severe oral-motor disabilities that did not allow substantial improvements. They all presented with severe involvement of multiple cranial nerves. Two of them still required respiratory support by tracheostomy.

After the age of 2 years, in most patients (14/16), swallowing assessment found a light but steady improvement in feeding. All oral feeders started eating foods requiring chewing ability and started dividing food taste during meals at some point of their life, at a median age for both abilities of 6 years (maximum age: 12 years). A mature chewing pattern and a safe swallowing function for all textures was observed in less than half (6/14), acquired at a median age of 8.5 years. Considering the whole cohort, the percentage of children who reached a mature chewing pattern was 43% (7/16). For them, the swallowing process —including oral preparatory, oral transit, pharyngeal and oesophageal phases— during the assumption of hard solid foods was safe and properly performed. Children with still immature chewing pattern were able to eat soft solid food textures. An adequate oral-sensory processing was obtained by all oral feeders and was reached at a median age of 5.5 years. Three children systematically required extra time for completing the meal (from 20 to over 60 min), although less than previously needed, while the others were able to timely complete the meal at a median age of 5 years. Vomiting, gagging or splitting with certain textures were reported by parents during assessments to be slightly more frequent before the year of 5 than after. The parental concern regarding children eating slightly decreased with age as well as the compensatory strategies used to complete meals. Slightly more than half of the whole cohort reached an adequate meal time duration during scholar age.

## Discussion

This is the first study reporting longitudinal data on the development of feeding skills in CS, for whom an increasing long-life expectancy has been assessed by previous studies [12]. Therefore, our data add new insights into nutrition issues of CS predicting feeding trajectory among different ages.

The present report provides new data on the prevalence of feeding issues at birth and over time in CS. Moreover, by offering a picture of the evolution of feeding abilities in an unselected and relatively large CS cohort, the present data give a reference on the age at which feeding abilities could be reached, allowing quantifying the progression of the risk of tube feeding. As the percentages of major and minor clinical features of our cohort fit into the ones previously reported by the medical literature [9, 11, 13], we suppose that our findings may be useful for clinicians who manage CS patients.

All CS new-borns of our cohort experienced feeding issues and 60% required TEN. Neonatal suckling ability and coordination between sucking and swallowing was severely compromised. Therefore, in CS new-borns, we noticed that daily feeding remains challenging to manage. The involvement of multiple factors, including craniofacial anomalies [22], structural defects [22], neurological impairment [4] and prematurity [23] produce concerns and make feeding a complicated task in CS new-borns.

Our study confirms that in early life, CS patients may require tube feeding due to floppiness and major anatomical difficulties, occurring in 25% in the first 2 years. Specifically, during the first months of life, infants are considered “preferred nose breathers” and, if nasal obstruction occurs, it negatively affects nutrition ability and other multiple functions [24]. As reported in previous studies [17, 25], the presence of choanal atresia and stenosis (20% in our population) causes an interruption in breathing through the nasal passage, and therefore, it may preclude the coordination between sucking, swallowing and breathing. In addition, occurrence of cleft palate (27% in our population) affects sucking ability, which can be ineffective due to the negative pressure useful to extract milk from the breast or bottle becomes unattainable [17]. Again, laryngeal/tracheal and oesophageal malformations endanger the protection of the respiratory tract with a subsequent higher risk of infection and prolonged hospitalisation [26]. Precisely, tracheoesophageal fistula was detected in 13% of our population, and for these patients, oral feeding was considered unsafe, requiring nutritional support through the artificial pathway until surgery.

Our findings also suggest that the ability to start weaning, physiologically developed between 4 and 6 months [27], was not timely reached by all children of our cohort due to oral-motor dysfunction and sometimes risk of bolus aspiration. At this time, gagging, fatigue or emesis may also occur during meals, and consequently, parents may not be able to safely feed their child. Specifically, none of the patients included in the study timely started eating solid food requiring chewing ability nor had adequate taste division during meals at 10–12 months of life. At 24 months of life, no one developed a mature chewing pattern, and 100% of children had prolonged meal time duration, while the oral-sensory processing was adequate in 7% out of the whole population.

As previously reported [15, 28], our data confirm that feeding difficulties are a common phenomenon in the CS population (Table 2). Despite this, most CS children gradually improved over time, though with a wide interindividual variability.

Based on the collected data, we can generalise that in CS children not requiring tube feeding, the ability of eating foods, involving chewing, can be achieved at school age, after the acquisition of an adequate oral sensory processing. However, it seems that a mature chewing pattern with a variety of food textures was not achieved by more than half of CS children in our cohort and by any of patients who required artificial enteral nutrition.

Moreover, a subgroup of patients persists in tube feeding dependence for many years. Nevertheless, the percentages of children with tube feeding dependence (60% at birth) over time face a slow but steady decrease (from 33% at 6 months, 25% at 12 months, to 13% at school age) in tandem with the decreasing risk of aspiration. Based on these data, we can assume that children who face severe feeding and swallowing disabilities accompanied by laryngeal/tracheal and oesophageal malformations at birth may have the lowest outcome for many years. We also observed that an early lack of experience with oral intake resulted in failure or considerable reduction of oral sensory-motor skills development. Although it is fair to investigate which components act as adverse factors for oral feeding development in the first year of life, the weight of the interactions of each component is, however, difficult to isolate. We can rather assume that in some cases, there are unfavourable multifactorial interactions between organic and developmental variables.

To note, mental retardation (MR) is one of the unfavourable variable affecting feeding. Primary developmental factors and acquired postnatal damages occurring in this rare condition are predictive of poor intellectual outcome. Specifically, in CS, the intelligence quotients (IQ) range from near-normal to profound retardation [4]. In turn, low adaptive behaviour skills and motor impairments, also along with neurologic dysfunction, render the acquisition of feeding skills quite challenging [29].

Dobbelsteyn et al. found that cranial nerve dysfunction may be the major underlying factor contributing to persistent feeding and swallowing difficulties [27]. Our patients with persistent worse outcomes had, among the other features, multiple cranial nerve involvement leading to tongue movement abnormalities and severe aspiration. As first suggested by Blake et al., and discussed thereafter by Dijk et al., a multidisciplinary involvement may improve CS patient’s management [12, 30]. The multidisciplinary evaluations of CS patients may bring to light other negative factors reducing interest in eating, such as anosmia, a loss of sensation of the oral cavity and the inefficient chewing ability. Cranial nerve dysfunction (73% in our population) will be reflected also in weak lip strength, immature chewing or sucking ability and drooling, a decreased sense of taste associated with a diminished gag and cough reflex. Bolus (silent) aspiration, gastroesophageal reflux and impaired tongue movement may also occur. Again, inadequate nutrition and fatigue on feeding are signs of congenital heart diseases and, accordingly, the nutritional effort may not be directly proportional to the nutritional input [12].

As expected, CS grade-schoolers have fewer feeding issues, although with some exceptions [22, 28]. As previously mentioned, the majority of patients in our cohort faced a light but steady improvement of feeding and swallowing during years. The low percentage of long-term worse outcomes can be explained by timely detection of feeding issues and oral-motor treatments, which acted as key factors in achieving successful oral feeding at some point [28]. Early rehabilitation has therefore many benefits for affected children, as it helps to timely promote eating-skills development and acts as a support for the family maximising the quality of life [31]. It plays an extremely important role in feeding skill evolution and in tube feeding removal [32]. Our data demonstrate that CS children gradually and through a long rehabilitation process can become “oral feeders,” but it is essential to accompany them with proper rehabilitative management and prevention activities.

As established for the infant with neurological impairment, if a baby with CS faces delay controlling body segments or reaching and maintaining the sitting position, it is essential to ensure a correct posture during meals [33]. An optimal sitting posture is essential for the stability of pelvic girdle and spine, and for a good head control with a consequent facilitation of swallowing. Oral-motor treatments follow the child’s growth as he/she may face difficulties chewing solid foods or managing different textures. A child with CS may also present a small mouth or labial incompetence with consequent failure retaining bolus in the mouth. Speech language therapists may introduce specific passive or active exercises improving these functions. Maladaptive behaviours related to feeding, if noticed, have to be modified into a more appropriate functioning during mealtimes through specific behavioural interventions [34]. Multiple feeding and swallowing follow-up accompany the different stages of the child’s life to ensure on-going swallow safety and adequate nutrition throughout adulthood [35–37].

In conclusion, this article provides clinically useful information to paediatric disability experts suggesting that although feeding issue is almost constant in CS population, a slow and gradual development of feeding abilities is detectable in most cases over time. A timely SLP intervention can be directly related to oral intake availability and weaning from enteral nutrition, in specific cases. Therefore, a proper management of dysphagia and nutritional issue should be made early and during the entire life span of every CS patient.

## Limitation and future research

Notwithstanding the collected data are particularly relevant in terms of patient management, we need to take into consideration two main limitations of the study. While it is based on a relatively large cohort of cases considering this rare disorder, the number of participants remains relatively small and requires further validation by an independent study. Another limit is the retrospective design of the study, though we obtained a large amount of longitudinal valuable data in a single-centre cohort of patients with molecularly confirmed CS due to the retrospective nature of the study. In future researches, a further in-depth analysis of the multiple food aversion leading causes should be investigated through the use of standardized scales.

Supplementary table 1. Prevalence of CHARGE features linked to feeding and swallowing difficulties reported in the literature *(last 5 years)*.

## Supplementary Information

Below is the link to the electronic supplementary material.

Supplementary file1 (DOCX 31 KB)

## Data Availability

The data generated during the current study are available from the corresponding author on reasonable request.

## Abbreviations

CS: CHARGE syndrome
ICF: International Classification of Functioning, Disability and Health
NGT: Nasogastric tube
TEN: Total enteral nutrition
VFSS: Videofluoroscopic Swallow Study
WHO: World Health Organization
