# Supplementary material for: Predicting the clinical trajectory of feeding and swallowing abilities in CHARGE syndrome
Source: Eur J Pediatr. 2023 Feb 17;182(4):1869–77. doi: 10.1007/s00431-023-04841-4 (PMC10167171; doi:10.1007/s00431-023-04841-4)
Supplement: Supplementary file 1 — Supplementary file1 (DOCX 31 KB) [file 431_2023_4841_MOESM1_ESM.docx]

**Supplementary Table 1.** Prevalence of CHARGE features linked to feeding and swallowing difficulties reported in the literature *(last five years).*

| Features | % | | | | |
| --- | --- | --- | --- | --- | --- |
|  | ***Our cohort (n=16)***  **^CHD7+^** | ***Simpson et al 2021 (n=18) ^[35]^***  **^CHD7+^** | ***Cheng et al 2019 (n=9) ^[36]^***  **^CHD7+^** | ***Legendre et al 2017 (n=119) ^[37]^***  ***^CDH7+ or CHD7-^*** | ***Hale et al 2016 (n=16) ^[11]^***  ***^CDH7+ CHD7-^*** |
| *Choanal atresia/stenosis* | 19 | nr | 0 | 43 (49/114) | 44 25 |
| *Retardation of growth/development* | 81 growth  100 development | 50 growth  100 development | 89 | nr | 73 58 growth  100 development |
| *Cranial nerve dysfunction* | 75 | 72 | nr | 77 (84/115) | 100 80 |
| *Cleft palate* | 25 | 28 | nr | 18 (21/117) | 31 25 |
| *Oesophageal atresia and/or TEF* | 12 | 17 | nr | 20 (21/107) | 19 ^†^  17 ^†^ |
| *Tracheomalacia* | 6 | nr | nr | nr | nr |

Nr= not reported; TEF= tracheoesophageal fistula, ^†^ isolated TEF
